# Chemopreventive Properties and Toxicity of Kelulut Honey in* Sprague Dawley* Rats Induced with Azoxymethane

**DOI:** 10.1155/2016/4036926

**Published:** 2016-07-25

**Authors:** Latifah Saiful Yazan, Muhamad Firdaus Shyfiq Muhamad Zali, Razana Mohd Ali, Nurul Amira Zainal, Nurulaidah Esa, Sarah Sapuan, Yong Sze Ong, Yin Sim Tor, Banulata Gopalsamy, Fui Ling Voon, Sharifah Sakinah Syed Alwi

**Affiliations:** ^1^Department of Biomedical Science, Faculty of Medicine and Health Sciences, Universiti Putra Malaysia (UPM), 43400 Serdang, Selangor, Malaysia; ^2^Laboratory of Molecular Biomedicine, Institute of Bioscience, Universiti Putra Malaysia (UPM), 43400 Serdang, Selangor, Malaysia; ^3^Department of Pathology, Faculty of Medicine and Health Sciences, Universiti Putra Malaysia (UPM), 43400 Serdang, Selangor, Malaysia

## Abstract

*Ethnopharmacological Relevance*. Colon cancer has been a major problem worldwide. Kelulut honey (KH) is produced by the stingless bees from* Trigona* species and has strong antioxidant activities that could be one of the potential chemopreventive agents from natural resources.* Aim of This Study*. This study investigated the chemopreventive properties and toxicity of KH in Sprague Dawley rats induced with azoxymethane (AOM).* Material and Method*. Twenty-four male Sprague Dawley rats aged 5 weeks were divided into 4 groups: (G1) untreated group not induced with AOM, (G2) untreated group induced with AOM, (G3) treated group induced with AOM, and (G4) treated group not induced with AOM. Injection of AOM (15 mg/kg) was via intraperitoneal route once a week for two subsequent weeks. The treatment groups were given oral administration of KH (1183 mg/kg body weight) twice daily for 8 weeks.* Results*. Treatment with KH significantly reduced the total number of aberrant crypt foci (ACF) and aberrant crypts (AC) and crypt multiplicity. KH was not toxic to the animals since the level of blood profile parameters, liver enzymes, and kidney functions was in normal range.* Conclusions*. The current finding shows that KH has chemopreventive properties in rats induced with colorectal cancer and also was found not toxic towards the animals.

## 1. Introduction

Cancer has been a major problem worldwide. Although colon cancer has been ranked the fourth, the incidence is rising dramatically. Colon cancer ranks the first among males and third among females [1]. There are several treatment modalities for colon cancer such as chemotherapy, surgery, and radiotherapy. Nevertheless, chemotherapy has a few adverse effects such as neutropenia (reduction of leucocytes), diarrhea, and thromboembolic events (blockage of blood vessel) [2]. Hence, numerous researches have been carried out in finding new alternatives for management of colon cancer.

One of the alternative managements is via chemoprevention. Chemoprevention is defined as the use of natural or synthetic substances to reduce the risk of developing cancer or to reduce the chance of cancer to recur. Thus, it is important to inhibit the development of epigenetic and genetic alterations that are part of the process by which clonal proliferation of cells with abnormal genetic content cascades into dysplastic and later malignant cells [3]. In colon cancer, it is an alternative approach to reduce the mortality which involves long-term use of a variety of chemopreventive agents that can inhibit development of neoplasms in the large bowel [4].

Kelulut honey (KH) is produced by the stingless bees from* Trigona* species. The honey produced by this stingless bee is stored in clusters of small resin pots near their nest. Meanwhile, honey that is produced by normally found bee is stored in hexagonal-shaped combs [5]. KH is more diluted compared to other types of honey and it has distinct taste (sour like taste) as well as aroma [6]. Traditionally, KH is used for antiageing, enhancing libido and immune system, killing bacteria, treatment of bronchial phlegm, and relieving sore throat, cough, and cold [7]. KH has been proven to possess various pharmacological properties such as anti-inflammatory [8], antioxidant [5], antiageing [9], and antibacterial properties [10]. Since KH has strong antioxidant activities, it could be one of the potential chemopreventive agents from natural resources.

## 2. Materials and Methods

### 2.1. Chemicals/Test Materials

Azoxymethane was purchased from Aldrich Sigma (St. Louis, United States), hematoxylin and eosin stain was purchased from CellPath (Mochdre, UK), 10% formalin was purchased from Fisher Scientific (Loughborough, UK), and RCL2® was purchased from Alphaleys (Plaisir, France). Kelulut honey (KH) was provided by Marbawi Food Processing and Trading, Kuala Kangsar, Perak, Malaysia.

### 2.2. Experimental Animals

A total of 24 male* Sprague Dawley* rats with body weight of 180 to 220 g at the age of 5 weeks were used. Prior to the experiment, the rats were acclimatized a week with free access to food and water* ad libitum*. The animals were housed in cages (*n* = 3) under standard laboratory conditions with 12-hour dark/light cycle, at 20–24°C with 40–50% relative humidity. Ethic approval was obtained from the Institutional Animal Care and Use Committee (IACUC) of Universiti Putra Malaysia prior to the study (UPM/IACUC/FYP.2015/FPSK.003).

### 2.3. Experimental Procedures

The rats were divided into 4 groups (*n* = 6), which were (G1) negative control group (not induced with AOM, without treatment), (G2) positive control group (induced with AOM, without treatment), (G3) treatment group (induced with AOM, treated with KH (1183 mg/kg)), and (G4) toxicity group (not induced with AOM, treated with KH). After acclimatization, AOM (15 mg/kg) was injected intraperitoneally into the rats for two subsequent weeks, once per week, on the first day of each week. Next, KH was administered orally twice daily, in the morning and at night, for eight weeks. The body weight of the rats was recorded every three days. The dosage of KH was calculated based on human intake. Upon termination, the rats were fasted for 8 hours before blood collection. Prior to blood sampling, the rats were anesthetized with ketamine-xylazine (75 mg/kg : 5 mg/kg) by intraperitoneal injection. The blood sample (7 mL) was collected by cardiac puncture using a 26 G, 1/2^″^ needle (Terumo®, Belgium, Europe) into nonheparinized (5 mL) and EDTA-containing tube (2 mL) for biochemical (Roche Cobas, USA) and haematological (ABX Pentra 60, Japan) analyses, respectively. The blood collected in nonheparinized tube was then centrifuged at 10,000 rpm for 10 minutes to separate the serum. The organs (colon, liver, and kidney) were removed and weighed before being fixed in 10% formalin.

### 2.4. Determination of Incidence of Aberrant Crypt Foci and Crypt Multiplicity

The colon was processed by first dividing it into three parts, which are proximal colon (near the caecum), middle colon, and distal colon (towards the rectum). Each part was cut into strip and placed in a cassette. For liver, it was grossed from a lobe of the liver and placed into a cassette. For kidney, the organ was cut into half and placed in a cassette. Next, the organs that had been grossed were processed using an automated tissue processor (LEICA TP 1020, USA) where the machine runs dehydration, clearing, and impregnating process. The organs were being embedded in molten paraffin with appropriate molds to support the tissue during sectioning process. The blocks were trimmed at 16 *μ*m thickness and sectioned at thickness of 0.4 *μ*m using a microtome. The thin sections of the tissue samples were placed in a water bath and fished onto glass slides. The slides prepared will be dewaxed first before being stained with hematoxylin and eosin (H&E) followed by mounting with P-xylene-bis-pyridinium bromide (DPX) and observed under a light microscope. The total number of ACF per colon, total number of aberrant crypts (AC), and crypt multiplicity of each rat in all groups were calculated.

### 2.5. Statistical Analysis

The data were analysed by Statistical Package for Social Sciences (SPSS) version 21. One-way ANOVA analysis was performed followed by Duncan's test. The data was presented as mean ± SEM. Value of *p* < 0.05 was considered as significant.

## 3. Results

### 3.1. Effects of KH on Body Weight

**Figure 1:**
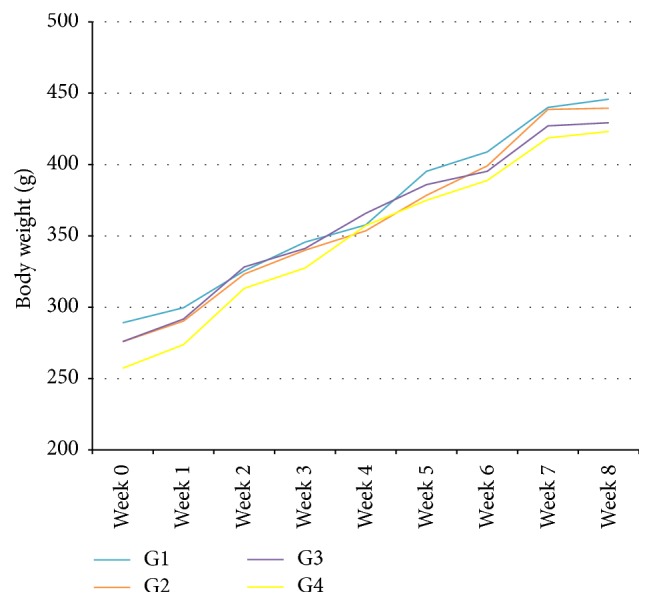
Effects of KH on the body weight of rats after 8 weeks of study. The data were analysed using one-way ANOVA and the values were expressed as mean ± SEM. Value of *p* < 0.05 was considered significant.

Figure 1 shows the body weight of all groups throughout the experiment. There was a gradual increase in the body weight throughout the 8 weeks of study. The highest body weight was noted in G2 followed by G1, G3, and G4. However, there was no significant difference in the body weight among all groups (*p* > 0.05).

### 3.2. Effects of KH on Blood Profile

The red blood cell (RBC), haemoglobin (Hb), packed cell volume (PCV), mean corpuscular volume (MCV), mean corpuscular haemoglobin concentration (MCHC), white blood cell (WBC), and thrombocytes level of rats in 4 different groups are summarized in Table 1. Data show no significant difference in the blood profile among all groups (*p* > 0.05).

### 3.3. Effects of KH on Liver Enzymes and Kidney Functions

Table 2 shows the liver enzymes and kidney function tests of rats in 4 different groups show no significant difference in all the parameters tested among all groups (*p* > 0.05).

### 3.4. Effects of KH on Colon Length

Table 3 shows the effects of KH on the length of the colon in all groups throughout the experiment. The highest colon length was noted in G1 followed by G3, G4, and G2. However, there was no significant difference in colon length among all groups (*p* > 0.05).

### 3.5. Effects of KH on Aberrant Crypt Foci

Table 4 shows the total ACF and total aberrant crypts (AC). The total number of ACF and AC and crypt multiplicity were significantly lower in G3 compared to G2 (*p* < 0.05).

### 3.6. Histological Classification of Aberrant Crypt Foci

**Figure 2:**
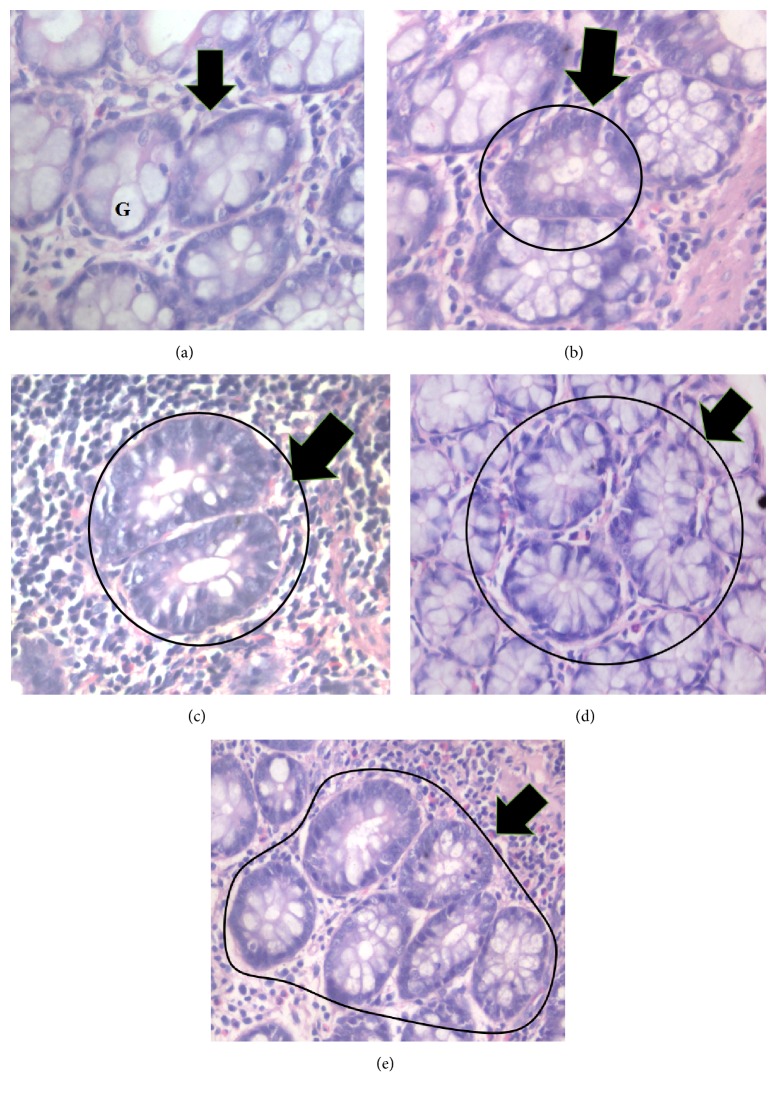
Photomicrograph of (a) normal crypt, (b) 1 crypt, (c) 2 crypts, (d) 3 crypts, and (e) ≥4 crypts stained with hematoxylin and eosin. Normal crypt is characterized by constant internal and diameter between the crypts and higher proportion of goblet cells (G), while ACF exhibit darker stain with reduced number of goblet cells (400x magnification).

ACF can be classified according to the number of crypts per foci, for example, 1 crypt, 2 crypts, 3 crypts, and ≥4 crypts per focus as being shown in Figure 2.

## 4. Discussion

This study was carried out to investigate the chemopreventive properties of KH towards* Sprague Dawley* rats induced with AOM. In this study, AOM was proven to induce ACF formation as reported by Bird [11]. In this model, ACF formation is used as biomarker to identify the progression of development of colon cancer. KH has chemopreventive properties against azoxymethane-induced colon cancer based on the reduction of total number of ACF, total number of AC, and crypt multiplicity.

Gribel and Pashinskiĭ [12] reported that honey possesses moderate anticolon cancer activity. Even though they used normal honey, Souza et al. [13] stated that there is only a slight difference in properties between honey produced by* Apis* bees and honey produced by stingless bees. It is believed that one of the components of KH that is responsible for its anticolon cancer properties is caffeic acid ester [14]. According to Xiang et al. [15], caffeic acid esters suppressed the transcriptional activity of *β*-catenin and hence can become an agent against colon cancer. Caffeic acid phenethyl ester (CAPE) is a phenolic antioxidant, which has biological and pharmacological functions that include immunoregulation, anti-inflammatory, antiviral, antibacterial, and antitumor activities [16]. CAPE has antiproliferative and apoptosis-inducing effect in various tumor cells* in vitro* and* in vivo* [17, 18]. According to Borrelli et al. [19], CAPE inhibited the development of AOM-induced ACF in the colon of rats.

In traditional usage, honey including KH is taken twice daily usually in the morning and at night. Based on the density, the daily intake of KH for human is 189.33 mg/kg. In this study, we were trying to mimic the way KH is being consumed by human. The dose of KH for rat consumption is calculated according to FDA dose translation formula. Thus, a rat received 1183 mg/kg bodyweight of KH.

Surprisingly, in this study, ACF were also found in the normal group. This could be due to the fact that normal body mutation also occurs. Mutation can occur in body because of several factors such as food, surroundings, and exposure to carcinogenesis agents [20].

KH was proven to be not toxic to the animals. There was no reduction in body weight. According to Peterson et al. [21], when there is a presence of toxicity, the body weight of the studied organism should be decreased. In addition, the levels of parameters for blood profile, liver enzymes, and kidney functions were in normal range. According to Wang et al. [22], abnormal values of blood profile, liver enzymes, and kidney function are an indication of toxicity.

In summary, KH has chemopreventive properties in rats induced with colon cancer by reducing the development of ACF, AC, and crypt multiplicity. KH was also to be found not toxic towards the animals.

## Figures and Tables

**Table 1:** Effects of KH on the blood profile of rats after 8 weeks of study. The data were analysed by using one-way ANOVA and the values are expressed as mean ± SEM. Value of *p* < 0.05 was considered significant.

Parameter	Group				
	Normal	1	2	3	4
RBC (×10^12^/L)	6.30–9.42	8.05 ± 0.07	8.31 ± 0.14	8.15 ± 0.12	8.40 ± 0.20
Hb (g/L)	10.0–17.0	151.16 ± 1.22	150.16 ± 1.49	153.16 ± 1.32	153.40 ± 2.94
PCV (L/L)	0.34–0.53	0.45 ± 0.0054	0.46 ± 0.0094	0.44 ± 0.0042	0.46 ± 0.0098
MCV (fL)	50.0–77.8	56.83 ± 0.40	55.50 ± 0.92	54.16 ± 0.47	55.25 ± 0.62
MCHC (g/L)	282.0–341.0	330.00 ± 2.72	325.83 ± 4.79	345.33 ± 2.04	329.60 ± 1.28
WBC (×10^9^/L)	2.9–20.9	7.45 ± 0.88	8.76 ± 0.36	9.32 ± 1.25	10.12 ± 0.95
Thrombocyte (×10^9^/L)	685.0–1436.0	642.00 ± 27.99	712.00 ± 31.43	663.50 ± 26.54	648.40 ± 52.00

**Table 2:** Effects of KH on the liver enzymes and kidney functions of rats after 8 weeks of study. The data were analysed by using one-way ANOVA and the values are expressed as mean ± SEM. Value of *p* < 0.05 was considered significant.

Parameter	Group				
	Normal	1	2	3	4
ALT (U/L)	17.50–32.30	39.36 ± 0.97	37.93 ± 1.60	41.08 ± 1.86	35.88 ± 2.16
ALP (U/L)	56.80–128.00	99.66 ± 5.07	89.16 ± 3.44	89.83 ± 2.61	96.20 ± 2.51
AST (U/L)	45.70–80.80	136.517 ± 9.70	145.85 ± 17.67	164.86 ± 16.42	150.78 ± 16.34
Creatinine (*µ*mol/L)	53.00–106.00	62.16 ± 0.60	66.33 ± 1.60	63.50 ± 1.38	65.60 ± 1.80
Urea (mmol/L)	1.80–7.10	4.83 ± 0.061	5.63 ± 0.19	5.51 ± 0.22	5.740 ± 0.20

**Table 3:** Effects of KH on the colon length of the rats after 8 weeks of study. The data were analysed by using one-way ANOVA and the values are expressed as mean ± SEM. Value of *p* < 0.05 was considered significant.

Group	Length (cm)
1	19.8667 ± 0.80691
2	17.6833 ± 1.30905
3	18.5167 ± 0.67103
4	17.7400 ± 0.73865

**Table 4:** Effects of KH on the incidence of azoxymethane-induced aberrant crypt foci of rats after 8 weeks of study. The data were analysed by using one-way ANOVA and the values are expressed as mean ± SEM. Value of *p* < 0.05 was considered significant.

Group	Total number of ACF	Number of crypts				Total aberrant crypts (AC)
		1	2	3	≥4	
Normal	3.67 ± 0.333	2.00 ± 0.577	1.67 ± 0.882	0	0	5.33 ± 1.202
Negative	29.33 ± 4.667	16.33 ± 2.963	6.33 ± 0.667	4.33 ± 0.882	2.67 ± 0.332	52.00 ± 7.371
KH-treated	10.67 ± 2.028	7.33 ± 1.333	3.00 ± 0.577	1.00 ± 0.577	0	15.00 ± 3.000

## References

[1] National Cancer Patient Registry—Colorectal Cancer http://www.crc.gov.my/reports/.

[2] André T., Boni C., Mounedji-Boudiaf L. (2004). Oxaliplatin, fluorouracil, and leucovorin as adjuvant treatment for colon cancer. *The New England Journal of Medicine*.

[3] Das D., Arber N., Jankowski J. A. (2007). Chemoprevention of colorectal cancer. *Digestion*.

[4] Jänne P. A., Mayer R. J. (2000). Chemoprevention of colorectal cancer. *The New England Journal of Medicine*.

[5] Kek S. P., Chin N. L., Yusof Y. A., Tan S. W., Chua L. S. (2014). Total phenolic contents and colour intensity of Malaysian honeys from the Apis spp. and Trigona spp. bees. *Agriculture and Agricultural Science Procedia*.

[6] Biluca F. C., Della Betta F., de Oliveira G. P. (2014). 5-HMF and carbohydrates content in stingless bee honey by CE before and after thermal treatment. *Food Chemistry*.

[7] Barakhbah S. A. S. A. (2007). Honey in the Malay tradition. *Malaysian Journal of Medical Sciences*.

[8] Sabir A., Tabbu C. R., Agustiono P., Sosroseno W. (2005). Histological analysis of rat dental pulp tissue capped with propolis. *Journal of Oral Science*.

[9] Afrouzan H., Bankova V., Tahmasebi G., Bigdeli M., Popova M. (2007). Comparison of gymnosperms and angiosperms plants on quality and quantity of propolis. *Pharmacognosy Magazine*.

[10] Zainol M. I., Mohd Yusoff K., Mohd Yusof M. Y. (2013). Antibacterial activity of selected Malaysian honey. *BMC Complementary and Alternative Medicine*.

[11] Bird R. P. (1987). Observation and quantification of aberrant crypts in the murine colon treated with a colon carcinogen: preliminary findings. *Cancer Letters*.

[12] Gribel N. V., Pashinskiĭ V. G. (1990). The antitumor properties of honey. *Voprosy Onkologii*.

[13] Souza B., Roubik D., Barth O. (2006). Composition of stingless bee honey: setting quality standards. *Interciencia*.

[14] Rao C. V., Desai D., Simi B., Kulkarni N., Amin S., Reddy B. S. (1993). Inhibitory effect of caffeic acid esters on azoxymethane-induced biochemical changes and aberrant crypt foci formation in rat colon. *Cancer Research*.

[15] Xiang D., Wang D., He Y. (2006). Caffeic acid phenethyl ester induces growth arrest and apoptosis of colon cancer cells via the *β*-catenin/T-cell factor signaling. *Anti-Cancer Drugs*.

[16] He Y.-J., Liu B.-H., Xiang D.-B., Qiao Z.-Y., Fu T., He Y.-H. (2006). Inhibitory effect of caffeic acid phenethyl ester on the growth of SW480 colorectal tumor cells involves *β*-catenin associated signaling pathway down-regulation. *World Journal of Gastroenterology*.

[17] Kuo H.-C., Kuo W.-H., Lee Y.-J., Lin W.-L., Chou F.-P., Tseng T.-H. (2006). Inhibitory effect of caffeic acid phenethyl ester on the growth of C6 glioma cells in vitro and in vivo. *Cancer Letters*.

[18] Oršolić N., Terzić S., Mihaljević Ž., Šver L., Bašić I. (2005). Effects of local administration of propolis and its polyphenolic compounds on tumor formation and growth. *Biological & Pharmaceutical Bulletin*.

[19] Borrelli F., Izzo A. A., Di Carlo G. (2002). Effect of a propolis extract and caffeic acid phenethyl ester on formation of aberrant crypt foci and tumors in the rat colon. *Fitoterapia*.

[20] Taylor R. W., Barron M. J., Borthwick G. M. (2003). Mitochondrial DNA mutations in human colonic crypt stem cells. *Journal of Clinical Investigation*.

[21] Peterson R. E., Seefeld M. D., Christian B. J., Potter C. L., Kelling C. K., Keesey R. E., Poland A., Kimbrough R. (1984). The wasting syndrome in 2, 3, 7, 8-tetrachlorodibenzo-p-dioxin toxicity: basic features and their interpretation. *Banbury Report: Biological Mechanisms of Dioxin Action*.

[22] Wang T. C., Su Y. P., Hsu T. Y., Yang C. C., Lin C. C. (2007). 28-Day oral toxicity study of the aqueous extract from spider brake (*Pteris multifida* Poiret) in rats. *Food and Chemical Toxicology*.

